# Impact of a Standardised Fetal Cardiac Screening Programme on Antenatal Detection Rates of Transposition of the Great Arteries

**DOI:** 10.1002/ajum.70004

**Published:** 2025-06-13

**Authors:** Alison Lee‐Tannock, Kate Jardine, Karen Eagleson, Jessica Suna, Kim Betts, Cameron Ward, Benjamin Auld, Alex Gooi

**Affiliations:** ^1^ Mater Research Institute, University of Queensland Brisbane Australia; ^2^ Mater Mothers Hospital Brisbane Australia; ^3^ Queensland Children's Hospital Brisbane Australia

**Keywords:** congenital heart disease, detection rates, fetal echocardiography, screening, transposition of the great arteries

## Abstract

**Background:**

Dextro‐transposition of the great arteries (d‐TGA) can be difficult to diagnose antenatally. Standardised cardiac screening protocols may improve detection rates.

**Aims:**

The objective of this study was to examine rates of antenatal diagnosis of d‐TGA in regional and metropolitan Brisbane over a 10‐year period and assess if a targeted antenatal screening education programme had an impact on detection rates.

**Materials and Methods:**

A retrospective cohort study was conducted. Data were collected for infants diagnosed with d‐TGA in Queensland between January 2008 and December 2017. Infants were divided into two cohorts to assess antenatal detection rates in both regional and metropolitan areas Queensland pre‐ and post implementation of a targeted sonographer education programme between 2008 and 2011.

**Results:**

A total of 126 infants were identified with a diagnosis of d‐TGA. The overall antenatal detection rate was 63.5% across the 10‐year study period. Prior to the educational intervention, the detection rate was 51% (2008–2011 *n* = 23/45), which increased significantly to 70% post educational intervention (2012–2017, *n* = 57/81) (*p* = 0.035). Regional Queensland (*n* = 60) detection rates increased from 44% to 63% (*p* = 0.192) and metropolitan (*n* = 66) detection rates increased from 60% to 76% (*p* = 0.24) post educational intervention.

**Conclusions:**

Rates of antenatal diagnosis of d‐TGA in Queensland compare favourably with internationally published rates, although difficulty in consistently diagnosing this congenital heart defect remains. A targeted educational programme of sonographers performing antenatal screening, particularly in regional areas, appears to increase rates of prenatal diagnosis and improve outcomes.

**Abbreviations**
Ao: aorta
CHD: congenital heart disease
d‐TGA: dextro‐transposition of the great arteries
IVS: intact ventricular septum
LV: left ventricle
MFM: Maternal Fetal Medicine
PA: pulmonary artery
RV: right ventricle
VSD: ventricular septal defect

**Summary**

- A simple targeted educational programme for sonographers can lead to an improvement in detection rates in transposition of the great arteries, a significant congenital heart defect (CHD).
- Our results suggest that continuing educational activities for sonographers in metropolitan and regional locations to improve detection rates in CHD are strongly recommended. This paper also suggests that a condensed training workshop is effective.

## 1 Introduction

Congenital heart disease (CHD) is a leading cause of infant mortality worldwide [1] but there is good evidence that reliable and accurate prenatal diagnosis reduces infant mortality, mainly by ensuring delivery takes place in a cardiac centre equipped to manage infants with complex cardiac abnormalities, particularly if emergency treatment and early cardiac surgical intervention are required [2, 3, 4, 5]. Prenatal detection rates for cardiac defects are variable and depend on the expertise of the sonographer or sonologist, type of cardiac abnormality, maternal habitus and gestation at which cardiac screening takes place. However, detection of outflow tract abnormalities is particularly challenging because the findings can be subtle and most babies with congenital heart defects are born to women with low‐risk pregnancies and will not be detected unless screened successfully [6, 7, 8, 9]. Transposition of the great arteries (d‐TGA), one of the most common cyanotic CHD with an incidence of 0.2–0.3 per thousand live births, [10, 11] is usually an isolated anomaly but is often missed prenatally [12].

Recent international studies have shown that the use of a protocol that ensures standardisation of specific fetal cardiac views combined with appropriate sonographer training is a cost‐effective way to increase antenatal detection rates of CHD [13, 14, 15, 16, 17, 18, 19, 20]. One study from Holland [21] demonstrated an improvement from 15.7% to 41% in d‐TGA detection rates after a standardised cardiac protocol and training for sonographers was instituted.

The aim of this study was to assess whether a standardised fetal heart screening protocol combined with appropriate sonographer education and training improved prenatal detection rates for d‐TGA in regional and metropolitan areas in Queensland.

## 2 Materials and Methods

Patient consent and ethics approval were obtained and approved by the Queensland's Research Hospital Ethics Committee (Queensland). Data were collected from the following sources: the Queensland Paediatric Cardiology Service (Queensland) cardiac surgical database and Queensland antenatal records.

This was a 10‐year retrospective study of d‐TGA detection rates for Queensland between 1st January 2008 and 31st December 2017. The starting date was chosen as we had complete data available from this date. The study was divided into two periods to differentiate between the pre/during and post educational programme detection rates. Epoch 1—1st January 2008 to 31st December 2011 (4 years) and Epoch 2—1st January 2012 to 31st December 2017 (6 years). Only infants with a confirmed postbirth diagnosis of an isolated d‐TGA were included in the study. Infants with complex d‐TGA, defined as having an additional significant cardiac abnormality, were excluded. Twelve cases which were diagnosed antenatally and had travelled to Queensland in utero for delivery were excluded.

All prenatal ultrasound screening throughout Queensland is performed by general and obstetric sonographers, who do not necessarily have specific training to identify cardiac anomalies. If a cardiac anomaly is suspected, the woman is then referred to a tertiary Maternal and Fetal Medicine (MFM) centre for detailed fetal echocardiography with specialist MFM sonographers and a paediatric cardiologist. Confirmed cases of CHD have follow‐up fetal echocardiography at 34–36 weeks gestation. Consequently, fetal echocardiography is usually performed at diagnosis and again at 34–36 weeks gestation in Queensland, and delivery is planned at a tertiary centre with paediatric cardiac services and surgery.

Between 2008 and 2011, a series of cardiac training seminars was conducted in five Queensland centres. These consisted of a full day of interactive teaching and live‐scanning workshops led by an experienced MFM sonographer (ALT) and a consultant paediatric cardiologist (AG). All attendees received credit points towards professional development.

Attendees were trained on methods to maximise image optimisation and the use of a specific fetal heart assessment protocol to assess the cardiac anatomy. This protocol detailed the following mandatory views:

1. The Four‐Chamber View.
2. The Outflow Tract Views: (a) right ventricle to pulmonary artery (RV‐PA); (b) left ventricle to aorta (LV‐Ao).
3. The Three‐Vessel Trachea View (3VTV).
4. The Aortic and Ductal Arch View (arrowhead view).

**FIGURE 1:**
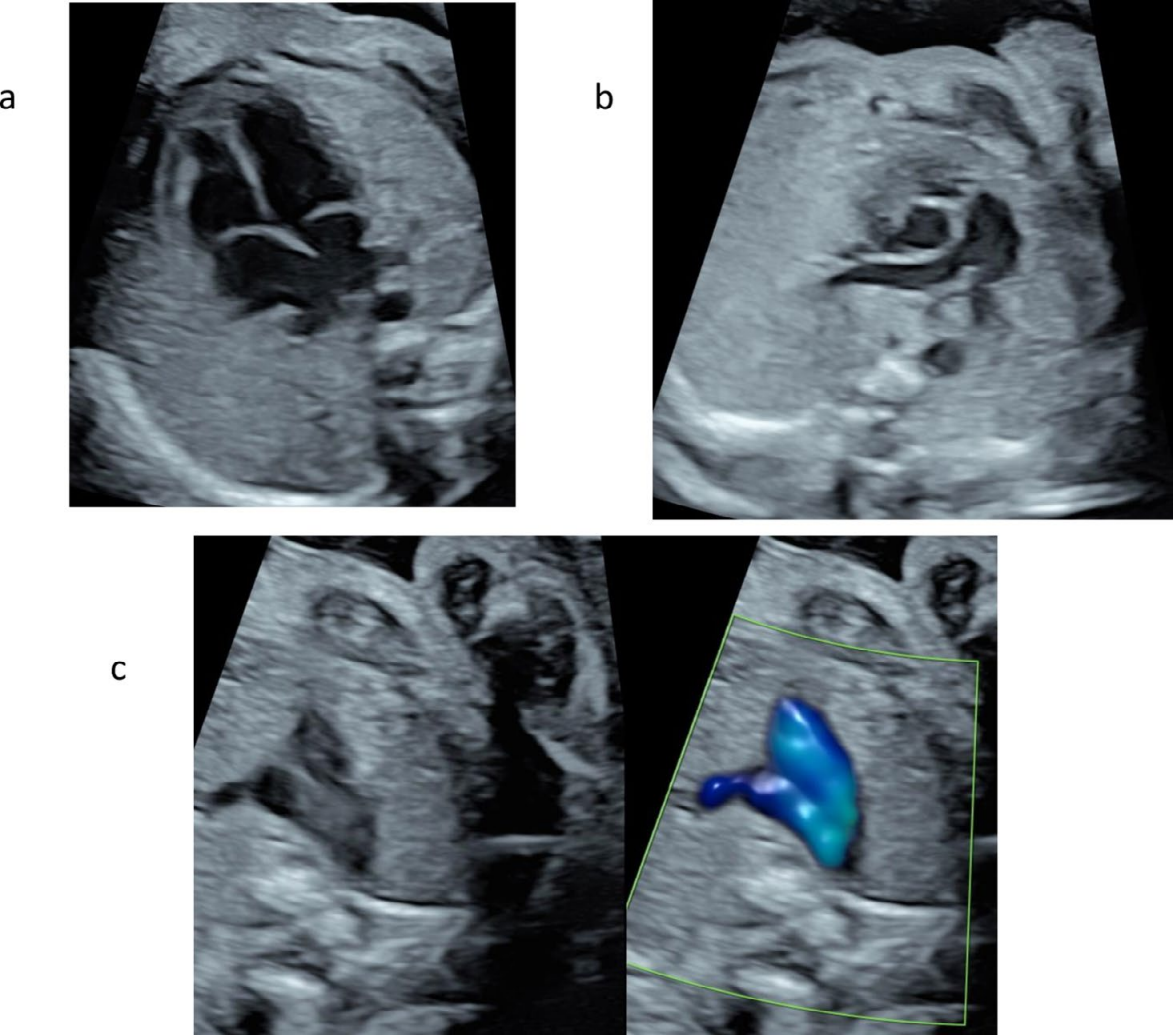
Normal axial fetal heart images. (a) 4C view. (b) 3VV. (c) Arrowhead view.

**FIGURE 2:**
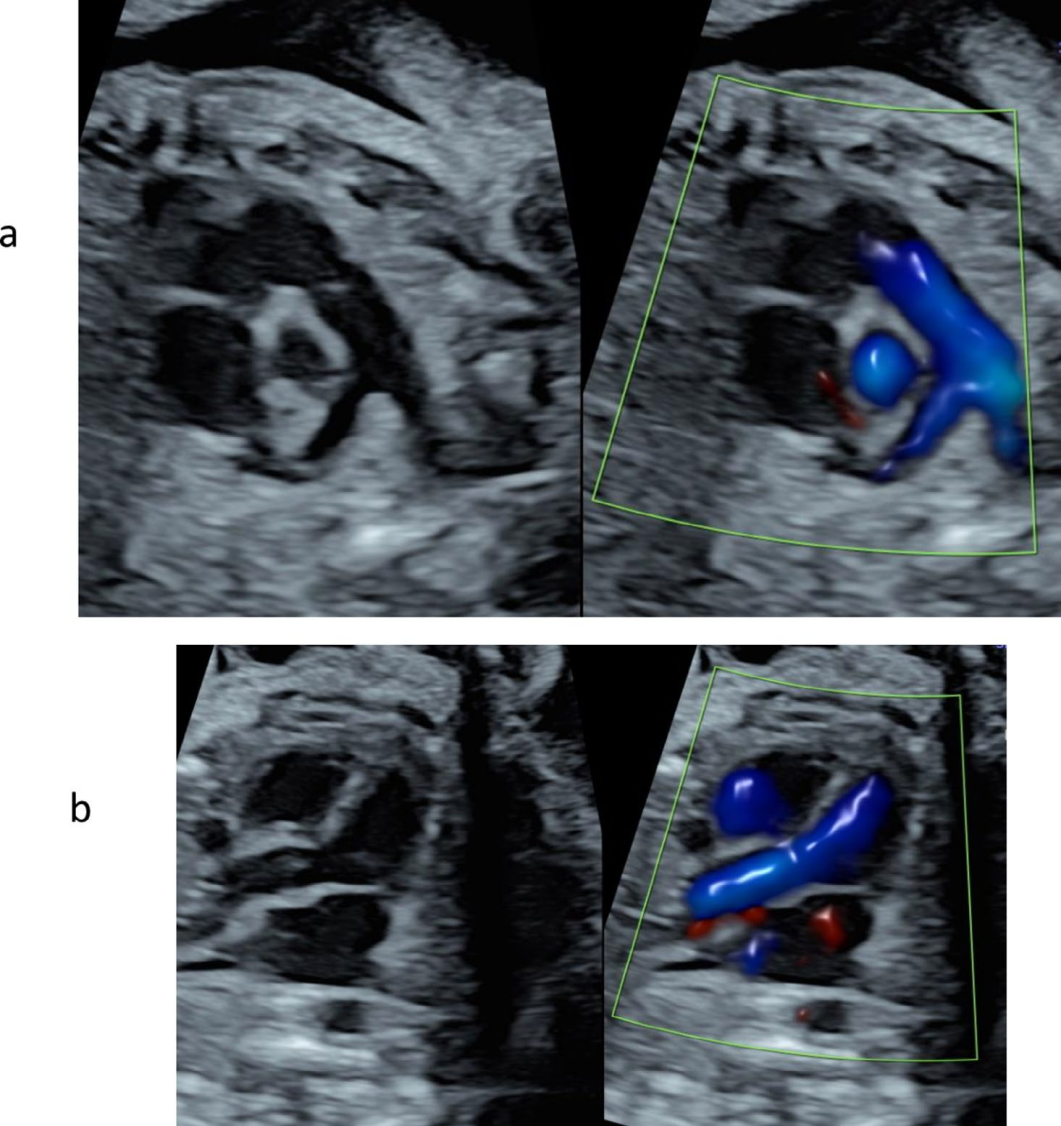
Normal outflow tract views. (a) RVOT (RV to PA). (b) LVOT (LV to Ao).

**FIGURE 3:**
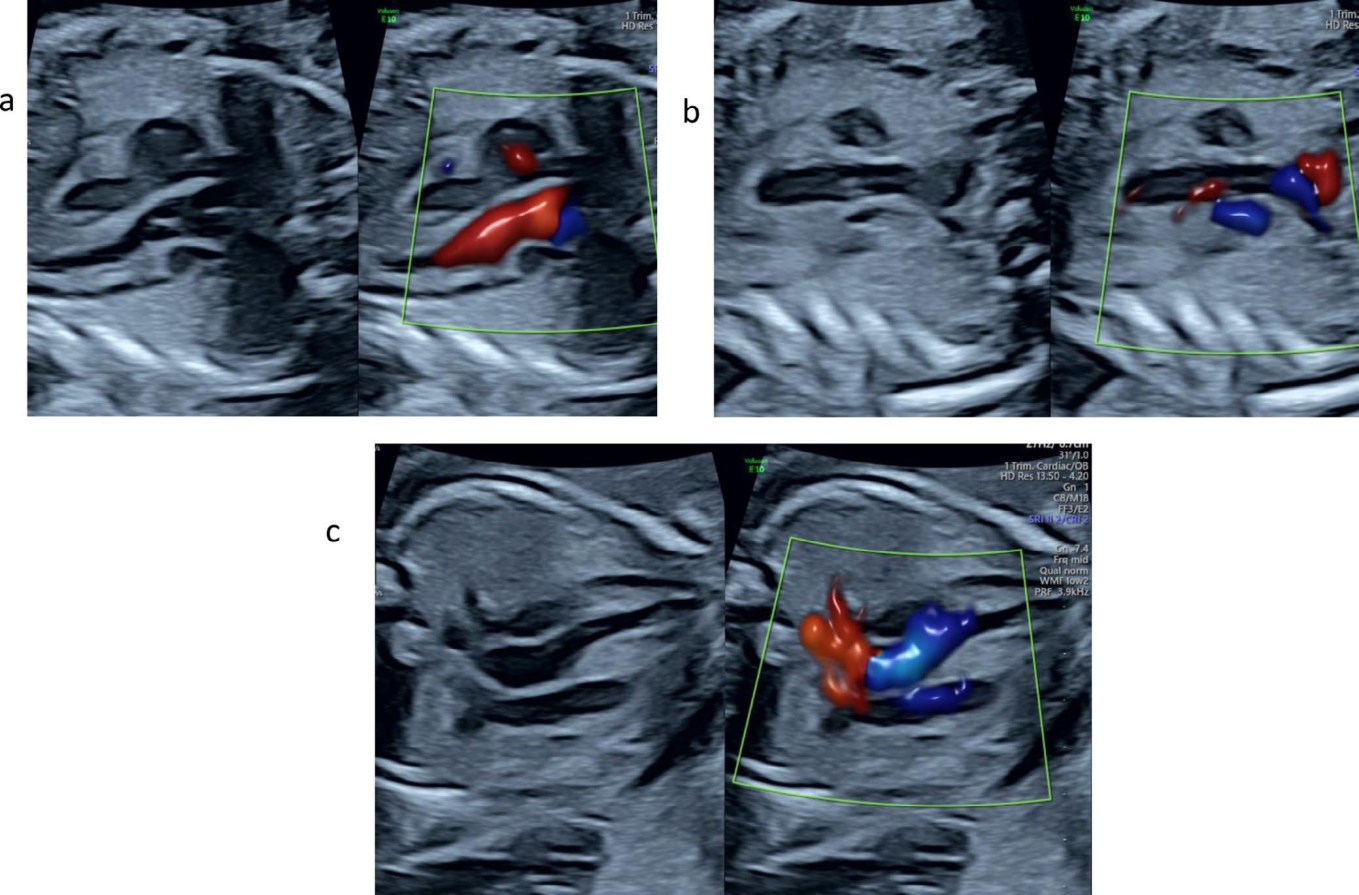
Outflow tract views with TGA. (a) LV to PA. (b) Abnormal 3VV. (c) Parallel outflow tracts.

Examples of the views required are shown in Figures 1 and 2. Cardiac images of cases of d‐TGA as well as those with more complex anatomy (Figure 3) were presented. Attendees were encouraged to extend the cardiac imaging to additional views (e.g., sagittal arch views) if the standard transverse views could not be obtained or appeared abnormal. A similar educational approach has been applied in many other international centres [15, 16].

### 2.1 Statistical Analysis

The antenatal detection rate was compared firstly between the two study periods (Epochs 1 and 2) and secondly between metropolitan and regional centres using the Student's *t*‐test. A *p* value of < 0.05 was considered to be statistically significant. All data were analysed using Stata version 14 (Statacorp, College Station, TX, USA).

## 3 Results

Over the whole study period, there were 126 neonates with confirmed d‐TGA. Of these, 80 infants (63.5%) were prenatally diagnosed with TGA. Although antenatal detection rates in metropolitan centres were higher compared with regional centres over the entire study period, this difference was not statistically significant (71% vs. 55%, *p* = 0.066). Overall antenatal detection rates significantly improved from 51% in the first epoch to 70% in Epoch 2 (*p* = 0.036).

In metropolitan centres, although antenatal detection rates increased from 60% (12/20) in Epoch 1 to 76% (35/46) in Epoch 2, this improvement was not statistically significant (*p* = 0.240). Similarly, in regional centres, although detection rates increased from 44% (11/25) in Epoch 1 to 63% (22/35) in Epoch 2, the difference remained nonsignificant (*p* = 0.192). (Table 1).

**TABLE 1:** Antenatal detection rates in Queensland July 2008 to December 2017 comparison overall and of metropolitan Queensland to regional Queensland.

	Antenatal detection 2008–2011, *n* (%)	Antenatal detection 2012–2017, *n* (%)	*p*
Whole cohort (*n* = 126)	23 (51%)	57 (70%)	0.036*
Metropolitan Brisbane (*n* = 66)	12 (60%)	35 (76%)	0.240
Regional Queensland (*n* = 60)	11 (44%)	22 (63%)	0.192

* Indicates statistically significant.

In the time before the training programme (Epoch 1), detection rates in metropolitan centres (60%) were higher than in regional centres (44%). This difference continued into Epoch 2 with improved detection rates in both metropolitan and regional centres (76% vs. 63%).

The majority of antenatal diagnoses of TGA (77.5%, 62/80) were made at the morphology scan (17–22 weeks gestation) whilst 22.5% (18/80) of cases were detected at 23–39 weeks gestation.

Reasons for late diagnosis (> 23 weeks) included delayed morphology scan (3/18) or missed diagnosis at morphology scan with subsequent detection at a subsequent scan for a noncardiac indication (13/18). The majority (67%, 12/18) of late diagnoses of d‐TGA occurred in regional centres.

## 4 Discussion

The key findings of this retrospective clinical review are firstly that overall detection rates of d‐TGA are higher in metropolitan versus regional centres and secondly, the implentation of a fetal cardiac education program resulted in an improvement in detection rates post implementation. Furthermore, the rise in detection rates was evident in both metropolitan as well as regional centres with a larger increase seen in regional centres.

This is likely due to the already high antenatal detection rates in metropolitan centres prior to the introduction of the educational and training program. This may be attributable to greater access of metropolitan sonographers to continuing education, a higher retention rate of staff trained in fetal cardiac scanning, and the comparative ease of access to tertiary MFM services for women where a CHD is suspected. Improved detection rates in regional Queensland are particularly important as antenatal detection has been shown to improve outcomes, especially in this CHD [11, 22, 23].

Our results are consistent with other studies published by van Velzen et al. [21] in the Netherlands and Ravi et al. [24] in Alberta, Canada. It illustrates that improvement can be achieved by a time‐efficient, targeted educational programme delivered by experienced clinicians.

Van Velzen et al. [21] demonstrated an improvement in detection rates of TGA from 15.7% to 41% after the institution of a nationwide screening programme. However, unlike ours, the Dutch screening protocol did not include the three‐vessel view, which may explain their much lower detection rates compared to our study. Importantly, the Dutch investigators also demonstrated an improvement in morbidity and mortality in the antenatally detected group, thus emphasising the need to improve antenatal detection rates. Furthermore, the same group [25] reported a marked rise in antenatal detection rates of d‐TGA to 85% after an improvement attributed to the introduction of the three‐vessel view assessing outflow tracts.

Ravi et al. [24] evaluated the trends in prenatal detection of TGA over a 13‐year period in Alberta, Canada. There were 127 cases over the study period, and detection rates improved from 14% (2003–2010) to 50% (2011–2013) to 77% (2014–2015). The investigators attributed their improved detection rates to improvements in screening of cardiac outflow tracts in routine obstetric ultrasound examinations along with updated obstetric ultrasound guidelines. Another study by Everwijn et al. [25] showed improvements in detection rates of TGA over an 8‐year period (2007–2015) from 63% to an average of 85% following the introduction of the three‐vessel view into routine screening.

Improvements in the detection of other cardiac abnormalities has also been reported in other countries following the introduction of a targeted training programme. Hunter et al. [15] in 2000 reported an improvement in detection rates of cardiac malformations in the north of England from only 17% in 1994 to 36% in 1996. Their training programme consisted of an initial 1‐day education programme followed by two follow‐up visits, 6 weeks and 12 months later. McBrien et al. [16] in a more recent publication showed an improvement in detection rates in Northern Ireland over a 5‐year period. They reported antenatal diagnosis of four‐chamber defects rose from 38% to 54% as did detection of outflow tract abnormalities from 8% to 21% following just 2.5 days of training over a 1‐year period. In comparison, our study showed significantly higher detection rates of this outflow tract defect following a much shorter training programme.

Interestingly, in our study, a substantial number of cases were detected after the routine morphology scan. Detection of CHD in the late second and third trimester has been previously reported [26] and demonstrates the importance of assessing the fetal heart in the third trimester when the patient is referred for a nonrelated fetal or maternal indication.

Several limitations were encountered in this study. Firstly, we were unable to obtain information from the death registry and postmortem records to ascertain whether there were any perinatal deaths during our study period that may have been attributed to undetected d‐TGA. Secondly, as our education programme was held over a 3‐year period, it is difficult to demarcate a clear ‘pre’ and ‘post’ intervention period.

In conclusion, our study demonstrates that the introduction of a standardised cardiac screening protocol and focused sonographer training improves antenatal detection rates of d‐TGA. This improvement was seen across both regional and metropolitan centres in Queensland. It is vital that detection rates of d‐TGA are high because they allow for planned delivery in a tertiary obstetric unit where tertiary neonatal intensive care and paediatric cardiology and cardiac surgery expertise is available for early intervention, including lifesaving balloon atrial septostomy [5, 23].

Further research to determine whether these improvements in antenatal detection rates of transposition of the great arteries have been maintained and/or continue to improve is planned. Antenatal detection of other major congenital heart defects is another area of interest for future research.

## Author Contributions

**Alison Lee‐Tannock:** conceptualization; writing – original draft; writing – review and editing; Methodology. **Kate Jardine:** investigation; writing – original draft; methodology. **Karen Eagleson:** validation. **Jessica Suna:** validation. **Kim Betts:** formal analysis; data curation. **Cameron Ward:** conceptualization; investigation; supervision. **Benjamin Auld:** writing – review and editing. **Alex Gooi:** conceptualization; investigation; writing – review and editing; supervision.

## Conflicts of Interest

The authors declare no conflicts of interest.
